# Influence of Different Capping Agents on the Structural, Optical, and Photocatalytic Degradation Efficiency of Magnetite (Fe_3_O_4_) Nanoparticles

**DOI:** 10.3390/nano13142067

**Published:** 2023-07-14

**Authors:** Thandi B. Mbuyazi, Peter A. Ajibade

**Affiliations:** School of Chemistry and Physics, University of KwaZulu-Natal, Private Bag X01, Scottsville, Pietermaritzburg 3209, South Africa; 216006335@stu.ukzn.ac.za

**Keywords:** iron oxide, nanoparticle, photocatalytic degradation, methylene blue, methyl orange

## Abstract

Octylamine (OTA), 1-dodecanethiol (DDT), and tri-n-octylphosphine (TOP) capped magnetite nanoparticles were prepared by co-precipitation method. Powder X-ray diffraction patterns confirmed inverse spinel crystalline phases for the as-prepared iron oxide nanoparticles. Transmission electron microscopic micrographs showed iron oxide nanoparticles with mean particle sizes of 2.1 nm for Fe_3_O_4_-OTA, 5.0 nm for Fe_3_O_4_-DDT, and 4.4 nm for Fe_3_O_4_-TOP. The energy bandgap of the iron oxide nanoparticles ranges from 2.25 eV to 2.76 eV. The iron oxide nanoparticles were used as photocatalysts for the degradation of methylene blue with an efficiency of 55.5%, 58.3%, and 66.7% for Fe_3_O_4_-OTA, Fe_3_O_4_-DDT, and Fe_3_O_4_-TOP, respectively, while for methyl orange the degradation efficiencies were 63.8%, 47.7%, and 74.1%, respectively. The results showed that tri-n-octylphosphine capped iron oxide nanoparticles are the most efficient iron oxide nano-photocatalysts for the degradation of both dyes. Scavenger studies show that electrons (e^−^) and hydroxy radicals (•OH) contribute significantly to the photocatalytic degradation reaction of both methylene blue and methyl orange using Fe_3_O_4_-TOP nanoparticles. The influence of the dye solution’s pH on the photocatalytic reaction reveals that a pH of 10 is the optimum for methylene blue degradation, whereas a pH of 2 is best for methyl orange photocatalytic degradation using the as-prepared iron oxide nano-photocatalyst. Recyclability studies revealed that the iron oxide photocatalysts can be recycled three times without losing their photocatalytic activity.

## 1. Introduction

Water contamination is currently a life-threatening issue and public health emergency that must be addressed promptly to improve global water quality and maintain a healthy ecosystem [1,2]. The excessive discharge of environmental contaminants like organic dyes has become a hinderance to the provision of potable water worldwide [3,4,5]. Organic dyes, with their complex chemical structures with high molecular weights, degrade slowly, are presumably mutagenic and carcinogenic, and have the capacity to block light penetration, hence reducing photosynthetic reactions. As a result, dye-containing effluents disrupt the natural balance of the surface water and significantly impact human health and aquatic ecosystems [6,7,8,9].

Magnetic metal oxide nanoparticles such as ZnO [10,11], CuO [12,13], SnO_2_ [14], TiO_2_ [15,16], MgO, and FeO [17,18] have been used as photocatalysts to degrade organic dyes and as adsorbents for removing heavy metal ions. Magnetic photocatalysts combine catalytic characteristics with magnetism, allowing the photocatalyst to be conveniently recovered from the treated solution using an external magnetic field. Magnetic iron oxide nanoparticles, among other forms of nanomaterials, offer unique properties such as abundance, good resistance to corrosion, low toxicity, and chemical and photochemical stability, making them an attractive material for photocatalysis [19,20,21,22]. However, magnetic iron oxide nanoparticles have high surface energies because of their high specific surface-to-volume ratios; therefore, they tend to aggregate to reduce the surface energy, which can change their adsorption ability and magnetic efficiency [23]. Consequently, it is critical to control the particle sizes and distributions of magnetic nanomaterials to prevent agglomeration.

Surfactant selection is of paramount importance in the preparation of magnetic nanoparticles to prevent their aggregation and create narrow particle size distributions with optimal shape and morphology. Numerous studies on the passivation of iron oxide nanoparticles using capping agents have been conducted. Sodipo et al. [24] studied the influence of five different capping agents on the morphology and magnetic properties of magnetite nanoparticles. Sodium citrate was the most effective capping agent, producing nearly spherical nanoparticles with the least agglomeration. Sharma et al. [25] prepared α-Fe_2_O_3_ nanoparticles using sodium citrate, polyvinyl pyrrolidone (PVP), and starch as capping agents. The incorporation of capping agents into nanoparticles has been shown to improve their activity and stability as photocatalysts against methylene blue with a maximum efficiency of 98.8% dye removal using PVP-capped α-Fe_2_O_3_.

Guidolin et al. [26] prepared Fe_3_O_4_ nanoparticles by a sol–gel citrate–nitrate method. The magnetite nanoparticles degraded 93.4% of methylene blue after 210 min when 2250 mg·L^−1^ of the nanoparticles was used. Rivera et al. [27] investigated the Fenton-like process for methylene blue dye degradation utilizing Fe_3_O_4_ nanoparticles prepared by the electrochemical synthesis method. The results showed that complete degradation can be achieved using 2 g·L^−1^ of Fe_3_O_4_ nanoparticles at a concentration of 100 mg/L MB in an acidic medium. Modrogan et al. [28] evaluated the photocatalytic performance of the Fe_3_O_4_/PVA composite against methyl orange in the presence of hydrogen peroxide under UV light. Various reaction parameters, such as composite dosage, pH, and the amount of hydrogen peroxide (H_2_O_2_), were studied. The results indicated that the level of dye degradation is significantly dependent on the amount of H_2_O_2_ in the reaction medium. León-Flores et al. [29] reported the green synthesis of Fe_3_O_4_ nanoparticles, using the *Coffea arabica* L. extract to degrade methyl orange dye under UV light irradiation, and that 80% of the dye was degraded using 10 mg of the nanoparticles in 15 mL of dye solution. Moreover, studies have shown that the morphology and phase composition of iron oxide nanoparticles influences their photocatalytic efficiency [30].

In this study, we report the structural and optical studies of octylamine (OTA), 1-dodecanethiol (DDT), and tri-n-octylphosphine (TOP) capped iron oxide nanoparticles. The as-prepared iron oxide nanoparticles’ potentials as photocatalysts were evaluated for the degradation of methylene blue and methyl orange dyes under visible light irradiation. The photodegradation kinetics, the influence of pH, scavengers, and photostability were also evaluated.

## 2. Materials and Methods

### 2.1. Materials

FeCl_2_·4H_2_O, Fe_2_(SO_4_)_3_∙H_2_O, 25% ammonia solution, ethanol, octylamine, 1-dodecanethiol, trioctylphosphine, methylene blue, methyl orange, ethylenediamine tetraacetic acid disodium, ascorbic acid, silver nitrate, isopropanol, hydrochloric acid, and sodium hydroxide were purchased from Merck (Darmstadt, Germany). All the reagents were used as purchased without further purification.

### 2.2. Synthesis of Iron Oxide Nanoparticles

Iron oxide nanoparticles were prepared using the co-precipitation approach [31]. The experiment was conducted in a nitrogen flow. FeCl_2_·4H_2_O (0.2485 g, 0.00125 mol) and Fe_2_(SO_4_)_3_∙H_2_O (1.0445 g, 0.0025 mol) were dissolved in 100 mL of distilled water and heated to 80 °C. An amount of 15 mL of 25% ammonia solution was added to adjust the pH of the reaction mixture to 11. After 30 min, octylamine (OTA) was introduced, and the temperature was maintained at 80 °C and the reaction was further stirred for 1 h. The nanoparticles were then centrifuged at 3500 rpm and washed several times to remove the excess capping agent and unreacted materials. To evaluate the effect of capping agents, the same procedure was carried out with trioctylphosphine (TOP) and 1-dodecathiol (DDT).

### 2.3. Characterization

FTIR spectra were recorded using a Cary 630 FTIR spectrometer, and absorption spectra were recorded using a Perkin Elmer lambda 25 UV-Vis spectrophotometer. The dye degradation experiments were conducted using a 70W high-pressure mercury lamp. The morphology of the nanoparticles was determined using a transmission electron microscopy (TEM) image taken with a Japan Electrical Optical Laboratories (JEOL) JEM-1400 electron microscope equipped with Gatan digital microgram software. ImageJ analysis software was used to measure the particle size distribution.

### 2.4. Adsorption of Methylene Blue (MB) and Methyl Orange (MO) by Iron Oxide Nanoparticles

The batch adsorption efficiency of Fe_3_O_4_ nanoparticles was measured using methylene blue (MB) and methyl orange (MO) as model dyes. Typically, 50 mg of Fe_3_O_4_ nanoparticles were dispersed in 40 mL of 10 mg/L of dye solution and the mixture was stirred in the dark for 75 min. An aliquot was withdrawn from each flask at 15 min time intervals and was analyzed with a UV-visible spectrometer to determine dye removal. The adsorption capacity, Q (mg/g), of the adsorbent was calculated using Equation (1):

(1) $$Q=\left(\frac{C_{0}-C_{t}}{W}\right)V$$

where C_0_ is the initial concentration of dye (mg L^−1^), C_t_ is the concentration of dye (mg L^−1^) in solution at a time ‘t’, V is the volume (L) of the dye solution, and W is the mass (g) of the adsorbent used.

### 2.5. Photocatalytic Experiment

Methylene blue (MB) and methyl orange (MO) dyes were used to investigate the dye degradation performance of iron oxide nanoparticles. In a typical method, a stock solution is prepared by dissolving 10 mg each of MB and MO in 1000 mL of distilled water. A total of 40 mg of iron oxide nanoparticles were dispersed in 40 mL of dye solution, sonicated for 60 min, and stirred in the dark for 60 min. The mixture was then irradiated with visible light and the photocatalytic degradation of the dye was evaluated using 4 mL of each mixed solution every 30 min. A UV-Vis spectrophotometer was used to evaluate the reaction progress by observing the absorbance maxima of the dyes. The degradation efficiency was calculated as:

(2) $$D=\frac{A_{0}-A_{t}}{A_{0}}\times 100$$

where ‘D’ denotes degradation efficiency (%), A_0_ refers to dye solution absorbance at ‘t’ zero, and A_t_ to dye solution absorbance after time ‘t’.

### 2.6. Effect of pH

The influence of pH on the degradation efficiency of MB and MO under visible light was examined over a pH range of 2 to 10. The pH of the dye solutions was changed using 0.1 M aqueous HCl and NaOH solutions.

### 2.7. Radical Scavenger Experiment

To identify the primary reactive species generated during the photocatalytic degradation of MB and MO, scavenging studies were conducted. To do this, isopropanol (IPA), ethylenediamine tetraacetic acid disodium (EDTA-Na_2_), ascorbic acid (AA), and silver nitrate (SN) were introduced to the reaction mixture to quench •OH, h^+^, •O_2_^−^, and e^−^, respectively. Then, 40 mg of the photocatalyst and 5 mL of each scavenger (10 mM) were added to 40 mL of dye solution (10 ppm) [32]. The experiment was carried out for 180 min under visible light and the respective absorbance spectra were taken to monitor the reaction progress.

## 3. Results

### 3.1. Structural and Morphological Studies of the Iron Oxide Nanoparticles

**Figure 1:**
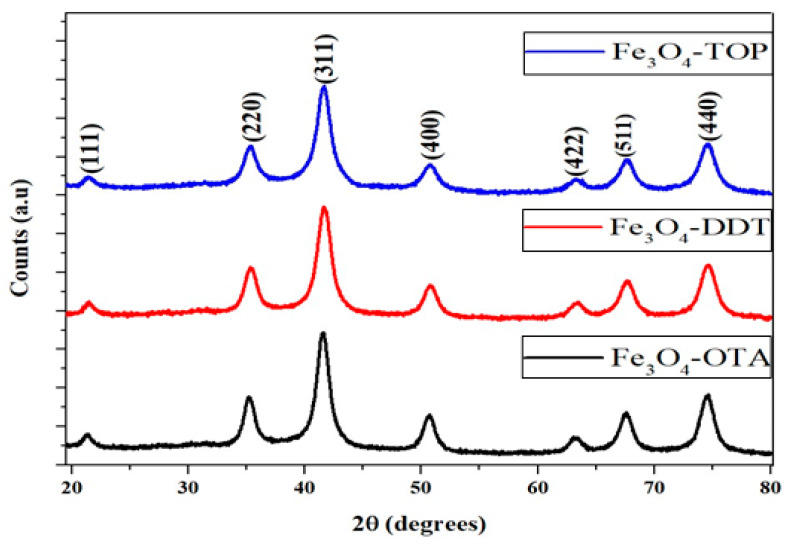
P-XRD diffraction patterns of OTA, DDT, and TOP-capped Fe_3_O_4_ nanoparticles.

Powder X-ray diffraction patterns of the as-prepared iron oxide nanoparticles are shown in Figure 1. All diffraction patterns were indexed to the single crystalline inverse spinel structure of magnetite with card No. 19-0629 [33,34]. The 2θ peaks are 21.45°, 35.30°, 41.59°, 50.67°, 63.14°, 67.68°, and 74.55°, corresponding to the (111), (220), (311), (400), (422), (511) and (440) planes of the cubic inverse spinel structure of Fe_3_O_4_. The crystal growth orientation preference is toward the (311) plane. The peaks are broad and well-defined which could be due to the small particle sizes and the crystalline nature of the as-prepared iron oxide nanoparticles. No other peaks corresponding to other phases were identified in the XRD patterns, indicating the formation of a pure Fe_3_O_4_ phase regardless of the capping agent used to prepare the iron oxide nanoparticles.

**Figure 2:**
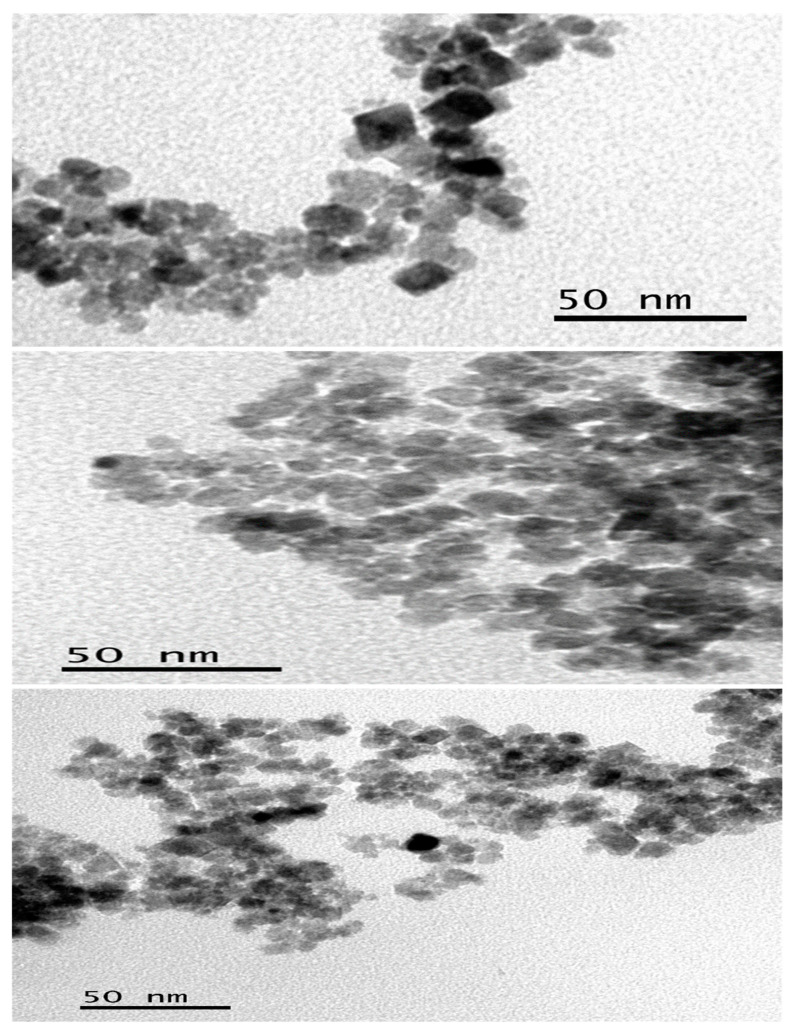
TEM images of OTA, DDT, and TOP-capped Fe_3_O_4_ nanoparticles.

TEM micrographs of the as-prepared iron oxide nanoparticles are shown in Figure 2. The TEM image of the OTA-capped iron oxide nanoparticles shows a mixture of square-like, oval, and quasi-spherical particles with an average size of 2.1 nm. The DDT-capped iron oxide nanoparticles also show agglomerated quasi-spherical particles with a mean particle size of 5.0 nm. The iron oxide nanoparticles prepared from TOP are also agglomerated with irregular shaped particles with a mean size of 4.4 nm (Figure S1). The observed agglomeration of the as-prepared nanoparticles may be attributed to the fact that they aggregate to reduce their high surface energy or to the dipole–dipole interactions of magnetic nanoparticles [24,35]. The use of capping agents resulted in more dispersed and well-defined shapes of nanoparticles compared to uncapped nanoparticles (Figure S2). Additionally, the use of different capping agents is observed to influence the as-prepared iron oxide nanoparticle sizes and shapes.

FTIR was used to confirm the interaction of the capping agents with iron oxide nanoparticles (Figure S3). Two weak peaks observed at 3387 and 3283 cm^−1^ in the OTA-capped iron oxide nanoparticle spectrum are attributed to the asymmetric and symmetric modes of NH_2_, respectively [36]. The asymmetric and symmetric C—H stretching vibrations of the alkyl chain of OTA are also observed at 2929 and 2833 cm^−1^. However, in the Fe_3_O_4_-OTA nanoparticles spectrum, these peaks are shifted to 2937 and 2905 cm^−1^. The C—N stretch of OTA appeared at 1477 cm^−1^, which is shifted to 1469 cm^−1^ in the Fe_3_O_4_-OTA spectrum. This indicates the presence of octylamine on the surface of iron oxide nanoparticles [37]. DDT shows sharp bands at 2913 cm^−1^ and 2849 cm^−1^, which are due to the asymmetric and symmetric C—H stretching modes, respectively [38,39]. These bands are blue-shifted by 16 and 8 cm^−1^, respectively, in the Fe_3_O_4_-DDT nanoparticles spectrum. The C—S stretching vibration at 723 cm^−1^ observed in the DDT spectrum is shifted to 826 cm^−1^ in the Fe_3_O_4_-DDT nanoparticles spectrum. The pure DDT also shows S—H stretching vibrational modes at 2564 cm^−1^ [40], which is shifted by 3 cm^−1^ in the Fe_3_O_4_-DDT nanoparticles spectrum. TOP exhibits C–P stretching vibrations at 1140 cm^−1^, while the bands at 2913 cm^−1^ and 2857 cm^−1^ are attributed to C—H stretching vibrational modes [41]. The C—H stretching vibrations were observed at 2928 cm^−1^ and 2856 cm^−1^ in the Fe_3_O_4_-TOP nanoparticles spectrum, while the C—P absorption band was observed at 1139 cm^−1^. The sharp bands at 552 cm^−1^ in iron oxide nanoparticles’ spectra are attributed to Fe—O vibration [42].

### 3.2. Optical Properties

**Figure 3:**
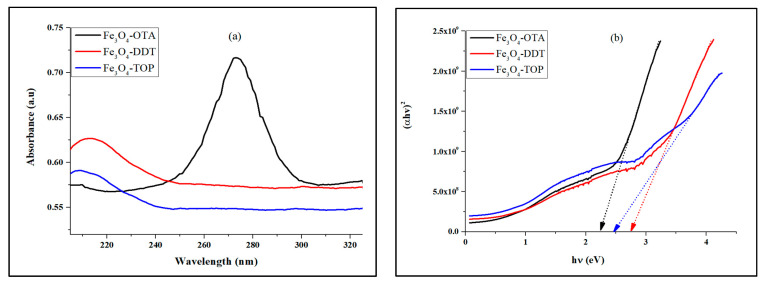
(**a**) UV-Vis spectra and (**b**) Tauc plots of iron OTA, DDT and TOP-capped iron oxide nanoparticles.

The optical absorption spectra of the as-prepared iron oxide nanoparticles in Figure 3a shows that Fe_3_O_4_–OTA, Fe_3_O_4_–DDT, and Fe_3_O_4_–TOP nanoparticles exhibit absorption peaks at 274, 210, and 209 nm, respectively. The peaks can be attributed to electrons moving from the oxygen atom to the *d*-metal orbital [43,44]. The optical band gaps estimated from Tauc plots (Figure 3b) were 2.25 eV for Fe_3_O_4_–OTA, 2.47 eV for Fe_3_O_4_–DDT, and 2.76 eV for Fe_3_O_4_–TOP. The band gap energy decreases with increasing capping agent chain length. The variation in absorption band edges and band gap energies may be due to the different morphologies of the nanoparticles. This indicates the modification of the valence band and conduction bands by the quantum confinement effect [45,46].

### 3.3. Adsorption Studies of Iron Oxide Nanoparticles

**Figure 4:**
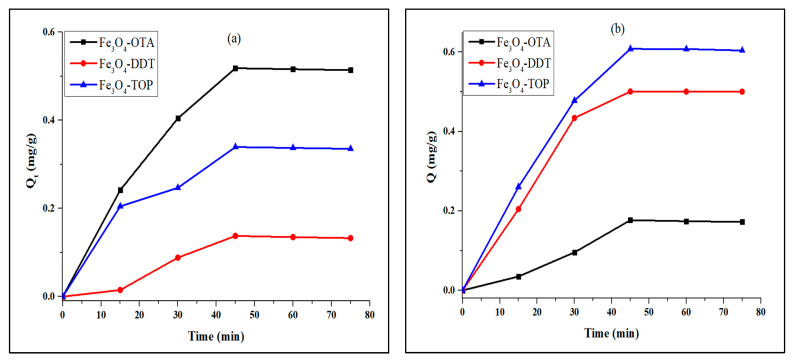
Time profile of (**a**) methylene blue and (**b**) methyl orange adsorption.

Adsorption is a surface phenomenon where pollutants transfer from a liquid phase to the surface of a solid phase via physical forces or chemical interactions [47]. Since photocatalytic degradation of contaminants takes place on the surface of the photocatalyst, the adsorption capacity test is crucial [48]. The Fe_3_O_4_ nano-photocatalysts reached equilibrium adsorption capacity for both methylene blue and methyl orange dyes at 45 min (Figure 4). The stability of Fe_3_O_4_ nanoparticles after 45 min demonstrates that the adsorption–desorption equilibrium has been attained.

### 3.4. Photocatalytic Degradation of Methylene Blue and Methyl Orange by Iron Oxide Nanoparticles

**Figure 5:**
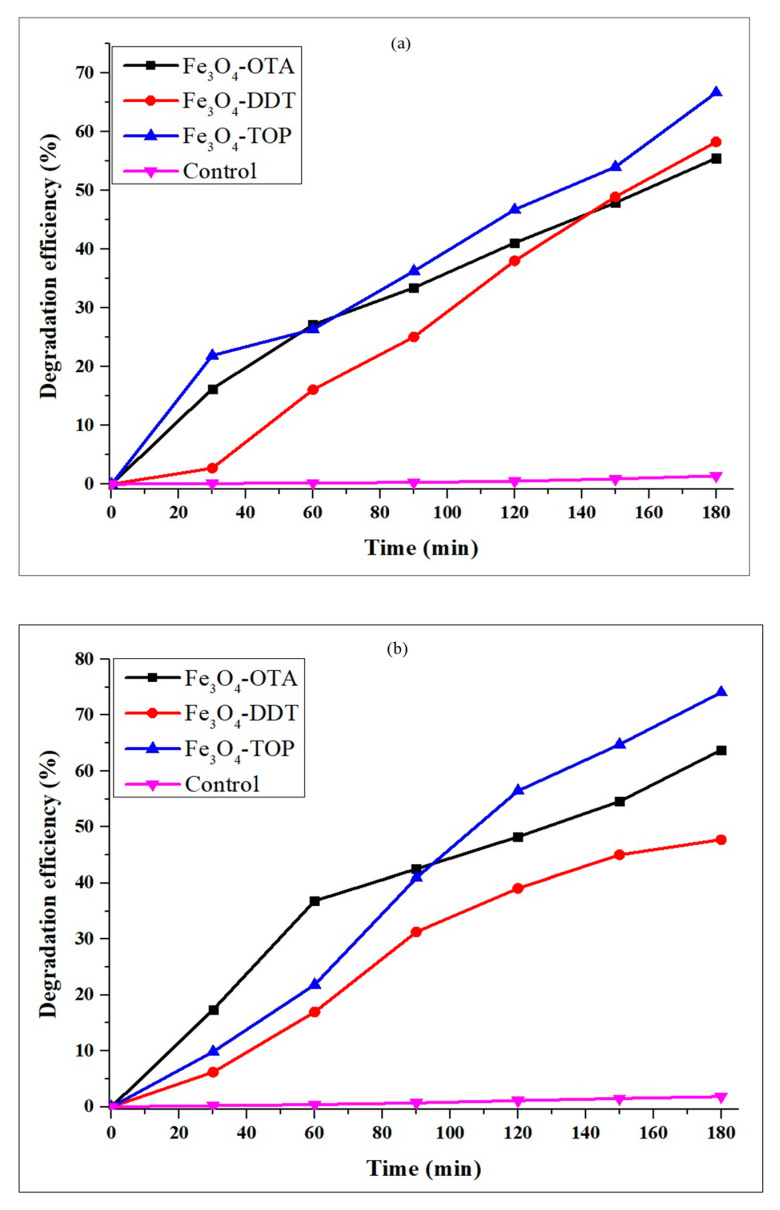
Degradation efficiency curves of (**a**) MB and (**b**) MO.

The photocatalytic activity of OTA, DDT and TOP-capped iron oxide nanoparticles was evaluated using methylene blue (MB) and methyl orange (MO) dyes as organic pollutants under visible light irradiation. The time-dependent absorption spectra of MB and MO show (Figures S4 and S5) a decrease in characteristic absorption peaks over time. In the absence of iron oxide nanoparticles, the degradation of the dyes was insignificant. However, the dyes degraded significantly in the presence of iron oxide nanoparticles, as indicated by the C_t_/C_0_ plot (Figure S6). The degradation efficiencies of MB by the iron oxide nanoparticles after 180 min irradiation are 55.5%, 58.3% and 66.7% by Fe_3_O_4_-OTA, Fe_3_O_4_-DDT, and Fe_3_O_4_-TOP, respectively (Figure 5a). The degradation efficiency is observed to increase with an increase in the chain length of the capping agent. The highest degradation efficiency of methylene blue obtained in this study is slightly higher than that of previous studies using γ–Fe_2_O_3_ nanoparticles (38%) [49], hematite iron oxide nanoparticles (51%) [50], and Fe_3_O_4_/ZnO nanocomposite (63%) [51]. Degradation efficiencies of 63.8%, 47.7%, and 74.1% were obtained for Fe_3_O_4_-OTA, Fe_3_O_4_-DDT, and Fe_3_O_4_-TOP, respectively, against MO dye (Figure 5b). OTA and TOP-capped iron oxide nanoparticles exhibit superior activity against MO compared to Fe_3_O_4_-DDT, and this could be due to Fe_3_O_4_-DDT’s large band gap. The nanoparticles’ agglomeration impacts their optical properties, which affects their capacity to absorb and disperse the incident radiation as well as their photocatalytic performance [52,53,54]. Therefore, the high photocatalytic degradation efficiency demonstrated by Fe_3_O_4_-TOP for both MB and MO dyes could be due to the Fe_3_O_4_-TOP nanoparticle morphology and light absorption capacity.

The photocatalytic degradation reaction of MB and MO follows a pseudo-first-order kinetic model as demonstrated by the linear correlation of the graphs of ln(C_t_/C_0_) over irradiation time. Equation (3) was used to calculate the rate constants.

(3) $$\ln\left(\frac{C_{t}}{C_{0}}\right)=-kt$$

The rate constants and correlation coefficients (R^2^) determined from the graphs in Figure S7 are shown in Table 1. The photodegradation rate constants of cationic dye are 4.28 × 10^−3^ min^−1^ for Fe_3_O_4_-OTA, 5.01 × 10^−3^ min^−1^ for Fe_3_O_4_-DDT, and 5.57 × 10^−3^ min^−1^ for Fe_3_O_4_-TOP, while the photodegradation rate constants of anionic dye are 0.00529 min^−1^ for Fe_3_O_4_-OTA, 3.96 × 10^−3^ min^−1^ for Fe_3_O_4_-DDT, and 7.76 × 10^−3^ min^−1^ for Fe_3_O_4_-TOP. The degradation rate constant of Fe_3_O_4_-DDT is low in the anionic dye but higher in the cationic dye, which is consistent with the degradation efficiency plot. The high correlation coefficient values show that the photodegradation of MB and MO dyes fits the pseudo-first-order kinetic model.

### 3.5. Effect of Scavengers on the Photocatalytic Degradation of Dyes

**Figure 6:**
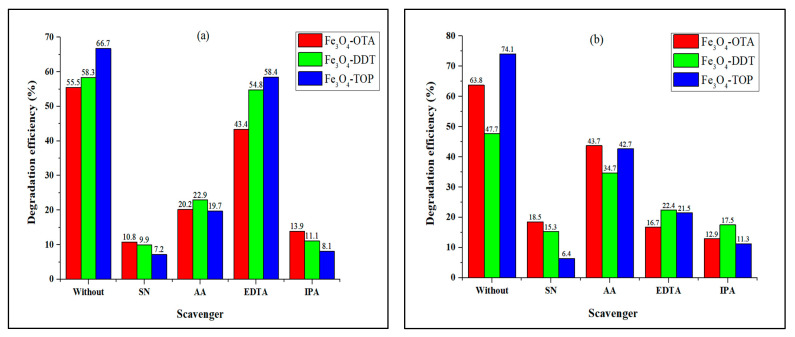
Effects of scavengers on the photodegradation of (**a**) MB and (**b**) MO by iron oxide nanoparticles.

A series of scavengers were added to the dye solutions to determine the active species in the photocatalytic degradation of methylene blue and methyl orange dyes by the as-prepared iron oxide nanoparticles. The results were contrasted to dye degradation efficiency obtained using only iron oxide nanoparticles, as shown in Figure 6. Silver nitrate (SN), isopropanol alcohol (IPA), ascorbic acid (AA), and ethylenediaminetetraacetic acid disodium salt (EDTA-2Na) were used to detect e^−^, •OH, •O_2_^−^, and h^+^ scavenging activity [55]. MB dye degradation efficiency by the as-prepared iron oxide nanoparticles decreases from 66.7–55.5% to 10.8–7.2% (SN), 22.9–19.7% (AA), 58.4–43.4% (EDTA), and 13.9–8.1% (IPA). The addition of scavengers also decreased the reaction rate constants as presented in Table 2 (Figure S8). This demonstrates that e^−^ and •OH are the main active species for the degradation of MB for all the as-prepared Fe_3_O_4_ nanoparticles. The results are consistent with previous studies using other photocatalysts [56,57]. However, the degradation efficiency of MO by Fe_3_O_4_–OTA decreases from 63.8% to 18.5% (SN), 43.7% (AA), 16.7% (EDTA), and 12.9% (IPA), whereas with Fe_3_O_4_–DDT the degradation efficiency decreases from 47.7% to 15.3% (SN), 34.7% (AA), 22.4% (EDTA), and 17.5% (IPA). The photocatalytic degradation efficiency of MO by Fe_3_O_4_–TOP decreases from 74.1% to 6.4% (SN), 42.7% (AA), 21.5% (EDTA), and 11.3% (IPA). In the presence of an ascorbic acid (AA) scavenger, Fe_3_O_4_-TOP exhibited a reaction rate of 1.58 × 10^−2^ min^−1^ (Figure S9), which is higher than the dye degradation rate without the scavenger; however, the degradation efficiency percentage remained low. The results indicate that e^−^ and •OH play significant roles in the degradation of MO using Fe_3_O_4_–DDT and Fe_3_O_4_–TOP, while •OH and h^+^ are observed to be the main active species with e^−^ acting as the secondary active species for Fe_3_O_4_–OTA.

### 3.6. Effect of pH

**Figure 7:**
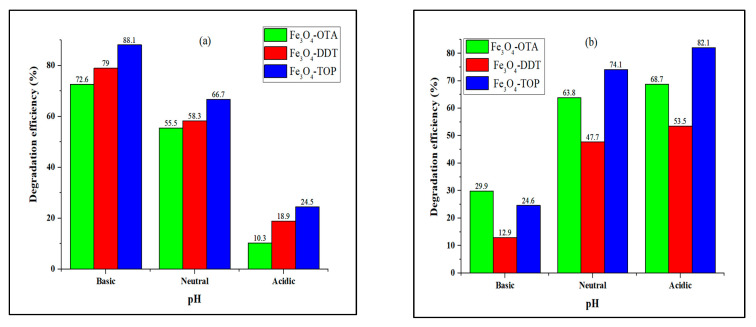
Effect of pH on (**a**) MB and (**b**) MO photocatalytic degradation by iron oxide nanoparticles.

Another variable that influences the efficiency of the catalyst charge surface, dye adsorption, and other photocatalytic reaction parameters is pH [58]. Figure 7 shows the results of a study on the photocatalytic degradation of MB and MO at various pH levels between 2 and 10 in the presence of iron oxide nanoparticles. For MB dye, low degradation efficiencies of 10.3–24.5% were obtained at pH 2 and high degradation efficiencies of 72.6% for Fe_3_O_4_-OTA, 79.0% for Fe_3_O_4_-DDT, and 88.1% for Fe_3_O_4_-TOP were obtained at pH 10. The higher degradation efficiency of MB degradation at pH 10 can be attributed to the enhanced adsorption of MB dye molecules on the negatively charged catalyst surface, resulting in a strong interaction between the photocatalyst and the generated radicals, resulting in rapid degradation [35]. In contrast, at pH 2, the existence of additional h^+^ in the reaction mixture made the catalyst surface more positive, inhibiting dye molecule adsorption due to columbic repulsion interactions, which resulted in a slower rate of radical generation and therefore degradation [59]. In contrast, the degradation efficiency of MO was observed to increase with decreasing pH, and the highest efficiency of 82.1% for Fe_3_O_4_-TOP was obtained at pH 2. The pH value has a major influence on the existing forms of methyl orange; in an acidic medium, it is in quinoid form, and it progressively transitioned to an azo structure as the pH of the solution increased [60,61]. In comparison to methyl orange in azo form, the quinoid structure is easier to degrade [62]. Hence, in an acidic medium, methyl orange dye degradation efficiency was higher.

### 3.7. Photostability Studies

**Figure 8:**
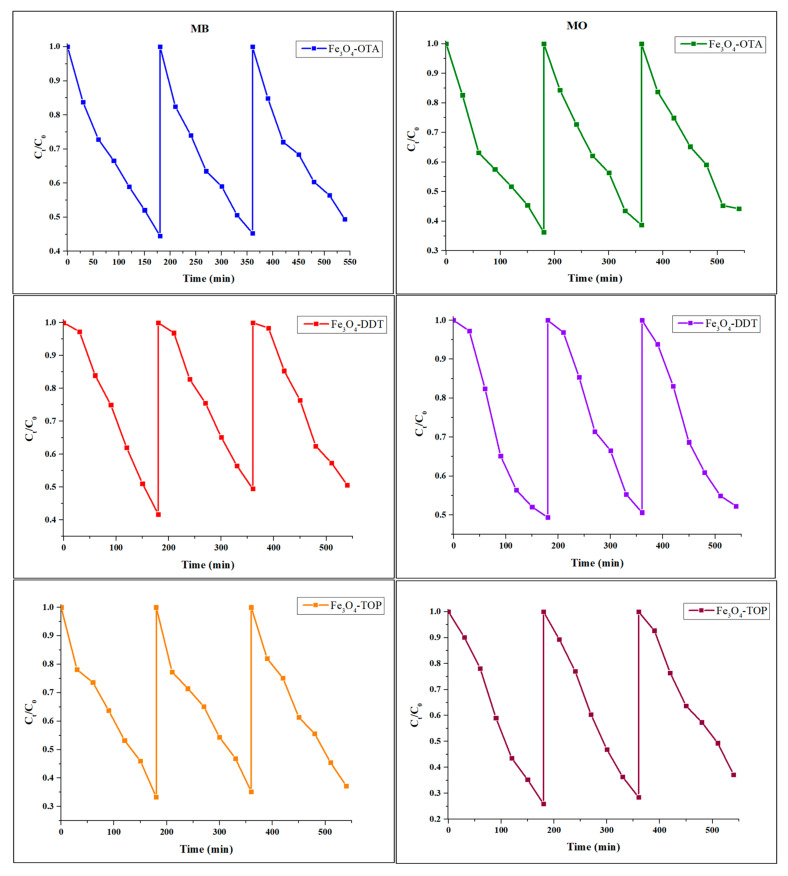
The recyclability of iron oxide nano-photocatalysts.

Photostability is a critical property of photocatalysts which makes photocatalysis a cost-effective, practical, and sustainable water treatment method [63]. Hence, recyclability studies were conducted to evaluate the iron oxide nanoparticles’ stability. Figure 8 demonstrates that the photodegradation efficiencies of the MB and MO dyes remained steady until the third cycle, with only a 0.8–11.3% decrease in efficiency over the three cycles. The decrease in photocatalytic degradation efficiency over the three cycles could be ascribed to either the build-up of intermediate products on the particle surface or nanoparticle loss during washing and filtration [64].

## 4. Conclusions

Iron oxide nanoparticles were prepared by co-precipitation method using three different capping agents to study the effect of capping agents on the morphological, optical, and photocatalytic properties of the as-prepared iron oxide nanoparticles. Powder X-ray diffraction patterns of the as-prepared iron oxide nanoparticles confirmed the cubic inverse spinel structure of Fe_3_O_4_. TEM images revealed Fe_3_O_4_-OTA nanoparticles are a mixture of square-like, oval, and quasi-spherical particles with an average particle size of 2.1 nm. Fe_3_O_4_-DDT nanoparticles were agglomerated quasi-spherical shapes with a mean size of 5.0 nm. The Fe_3_O_4_-TOP nanoparticles were also agglomerated and irregularly shaped with particle sizes of 4.4 nm. The results show that the use of different capping agents results in different particle sizes and shapes of the iron oxide nanoparticles. FTIR spectra confirmed the octylamine, 1-dodecanethiol, and tri-*n*-octylphosphine bond to the surface of the as-prepared iron oxide nanoparticles. Bandgap energies of 2.25, 2.47, and 2.76 eV were obtained for Fe_3_O_4_-OTA, Fe_3_O_4_-DDT, and Fe_3_O_4_-TOP, respectively. The results reveal that the bandgap energy can be tuned using different capping agents due to variations in the morphology of the nanoparticles obtained. The as-prepared iron oxide nanoparticles were used as photocatalysts for the degradation of MB and MO. The results showed that Fe_3_O_4_-TOP exhibited high degradation efficiency for the degradation of MB (66.7%) and MO (74.1%). The role of scavengers on the photocatalytic degradation efficiency was also investigated and e^−^ and •OH were found to be the major active species in the photodegradation reaction of MB for all Fe_3_O_4_ nanoparticles, respectively, whereas, for MO, •OH and h^+^ were found to be the major active species when using Fe_3_O_4_-OTA. The as-prepared Fe_3_O_4_ nano-photocatalysts are photostable and recyclable for the photocatalytic degradation of the organic dyes.

## Figures and Tables

**Table 1:** Photodegradation rate constants and correlation coefficients of MB and MO by iron oxide nanoparticles.

Dye	Catalyst	Degradation Efficiency (%)	Rate Constant (min^−1^)	R^2^
Methylene blue	Fe_3_O_4_-OTA	55.5	4.28 × 10^−3^	99.69 × 10^−2^
	Fe_3_O_4_-DDT	58.3	5.01 × 10^−3^	98.41 × 10^−2^
	Fe_3_O_4_-TOP	66.7	5.57 × 10^−3^	98.50 × 10^−2^
Methyl orange	Fe_3_O_4_-OTA	63.8	5.29 × 10^−3^	98.89 × 10^−2^
	Fe_3_O_4_-DDT	47.7	3.96 × 10^−3^	98.97 × 10^−2^
	Fe_3_O_4_-TOP	74.1	7.76 × 10^−3^	98.95 × 10^−2^

**Table 2:** Photodegradation rate constants of MB and MO by iron oxide nanoparticles in the presence of scavengers.

Dye	Catalyst	Rate Constant (min^−1^)			
		SN	AA	EDTA	IPA
Methylene blue	Fe_3_O_4_-OTA	6.15 × 10^−4^	1.84 × 10^−3^	3.18 × 10^−3^	8.54 × 10^−4^
	Fe_3_O_4_-DDT	1.12 × 10^−3^	1.36 × 10^−3^	4.68 × 10^−3^	6.84 × 10^−4^
	Fe_3_O_4_-TOP	4.39 × 10^−4^	9.08 × 10-4	4.78 × 10^−3^	5.62 × 10^−4^
Methyl orange	Fe_3_O_4_-OTA	1.26 × 10^−3^	3.38 × 10^−3^	9.97 × 10^−4^	7.66 × 10^−4^
	Fe_3_O_4_-DDT	9.53 × 10^−4^	2.51 × 10^−3^	1.42 × 10^−3^	1.18 × 10^−3^
	Fe_3_O_4_-TOP	3.52 × 10^−4^	1.58 × 10^−2^	1.47 × 10^−3^	6.61 × 10^−4^

## Data Availability

All research data are presented in the paper and the supplementary data.

## Supplementary Materials

The following supporting information can be downloaded at: https://www.mdpi.com/article/10.3390/nano13142067/s1, Figure S1. Size distribution histograms of OTA, DDT and TOP-capped iron oxide nanoparticles. Figure S2. TEM image of uncapped iron oxide nanoparticles. Figure S3. FTIR spectra overlay of octylamine, 1-dodecanethiol and tri-n-octylphosphine capped iron oxide nanoparticles. Figure S4. Absorption spectra of methylene blue (MB) over (a) Fe_3_O_4_-OTA, (b) Fe_3_O_4_-DDT and (c) Fe_3_O_4_-TOP nanoparticles. Figure S5. Absorption spectra of methyl orange (MO) over (a) Fe_3_O_4_-OTA, (b) Fe_3_O_4_-DDT and (c) Fe_3_O_4_-TOP nanoparticles. Figure S6. (a) MB and (b) MO C_t_/C_0_) versus irradiation time plots. Figure S7. Methylene blue (a) and methyl orange (b) photocatalytic degradation kinetics using iron oxide nanoparticles as photocatalysts. Figure S8. Photodegradation kinetics plots of MB by iron oxide nanoparticles in the presence of scavengers. Figure S9. Photodegradation kinetics plots of MO by iron oxide nanoparticles in the presence of scavengers.
