# Supplementary material for: Influence of Different Capping Agents on the Structural, Optical, and Photocatalytic Degradation Efficiency of Magnetite (Fe3O4) Nanoparticles
Source: Nanomaterials (Basel). 2023 Jul 14;13(14):2067. doi: 10.3390/nano13142067 (PMC10384526; doi:10.3390/nano13142067)
Supplement: Supplementary file 1 [file nanomaterials-13-02067-s001.zip › nanomaterials-2484446-supplementary.pdf]

Influence of different capping agents on structural, optical, and photocatalytic properties of iron oxide nanoparticles

Thandi B. Mbuyazi and Peter A. Ajibade*

School of Chemistry and Physics, University of KwaZulu-Natal, Private Bag X01, Scottsville, Pietermaritzburg 3209, South Africa

***Correspondence: ajibadep@ukzn.ac.za**

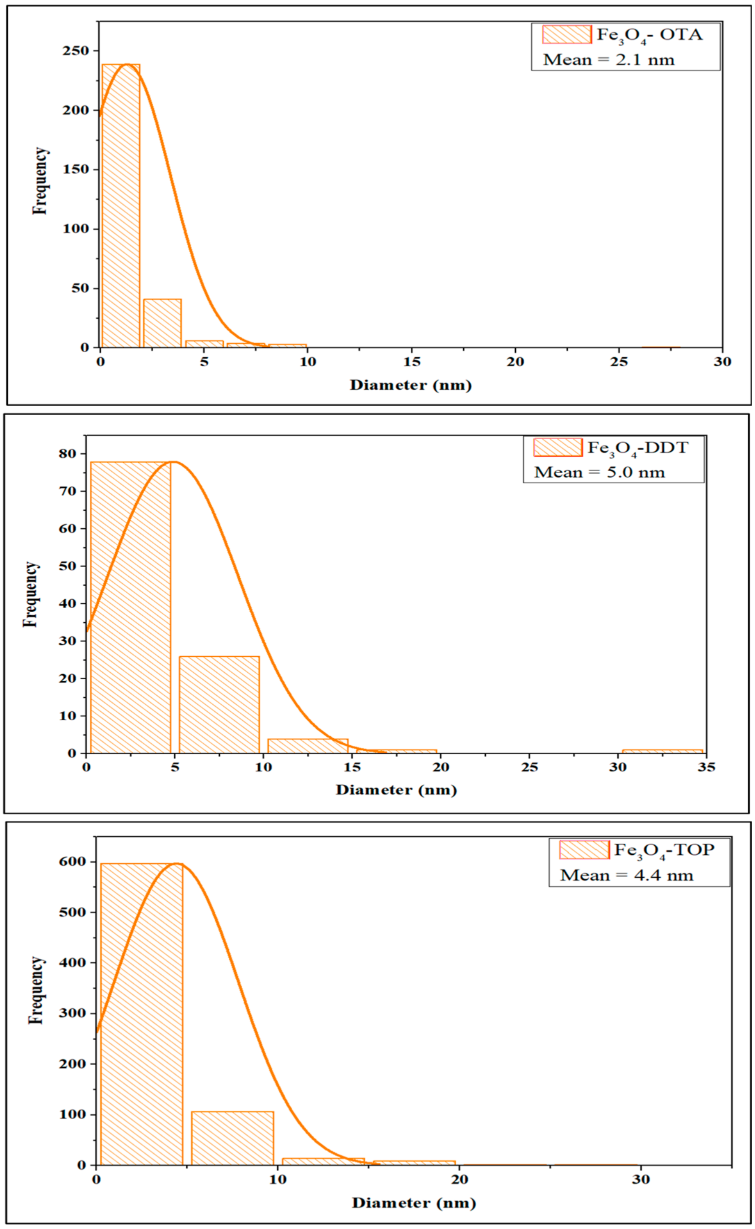

Figure S1. Size distribution histograms of OTA, DDT and TOP-capped iron oxide nanoparticles.

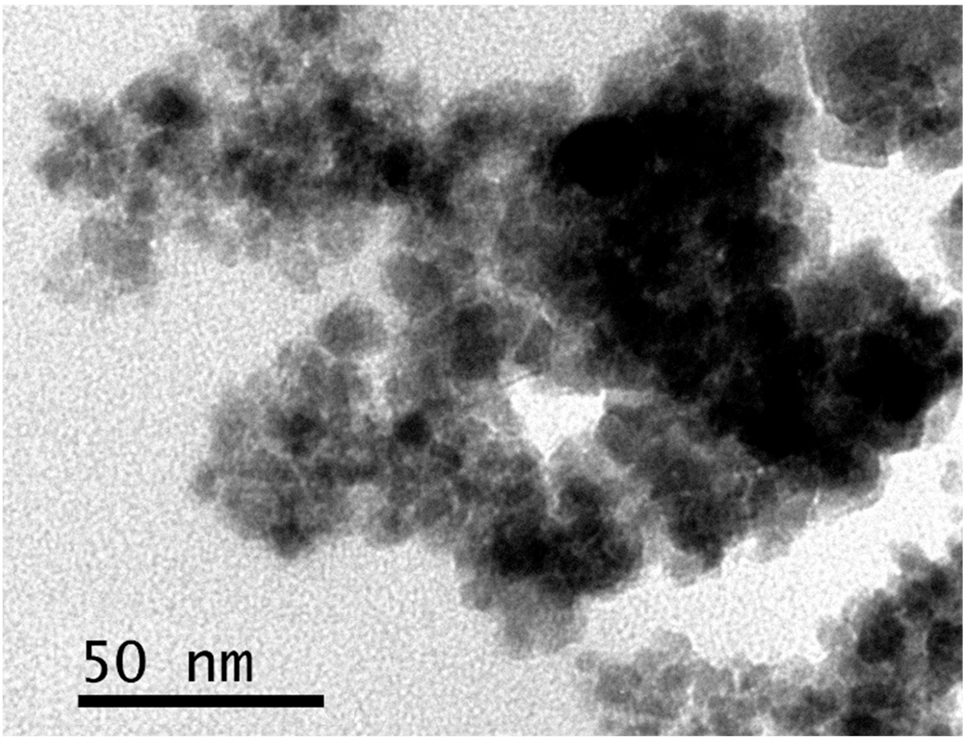

Figure S2. TEM image of uncapped iron oxide nanoparticles.

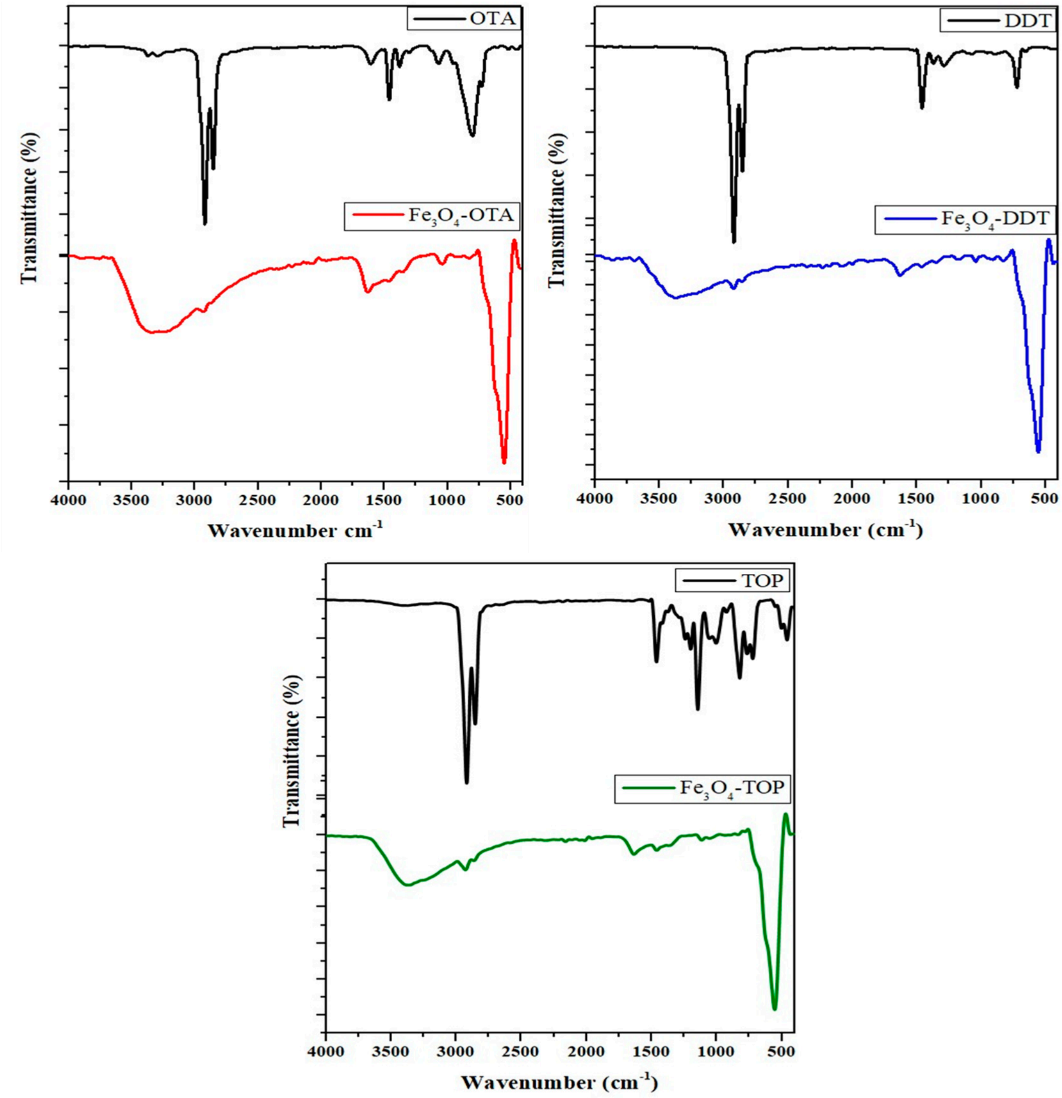

Figure S3. FTIR spectra overlay of octylamine, 1-dodecanethiol and tri-n-octylphosphine capped iron oxide nanoparticles.

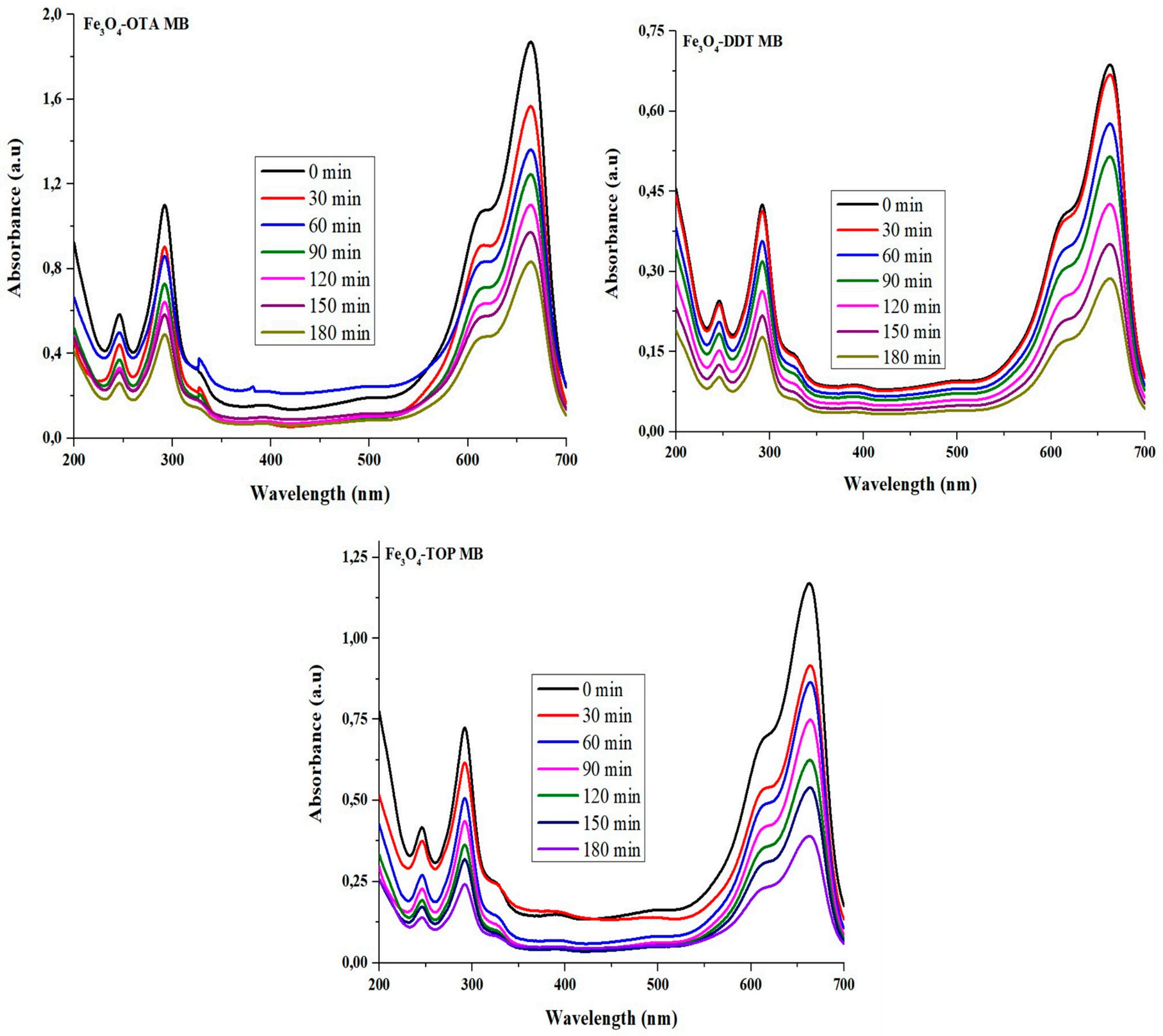

Figure S4. Absorption spectra of methylene blue (MB) over (a) Fe_3O_4-OTA, (b) Fe_3O_4-DDT and (c) Fe_3O_4-TOP nanoparticles

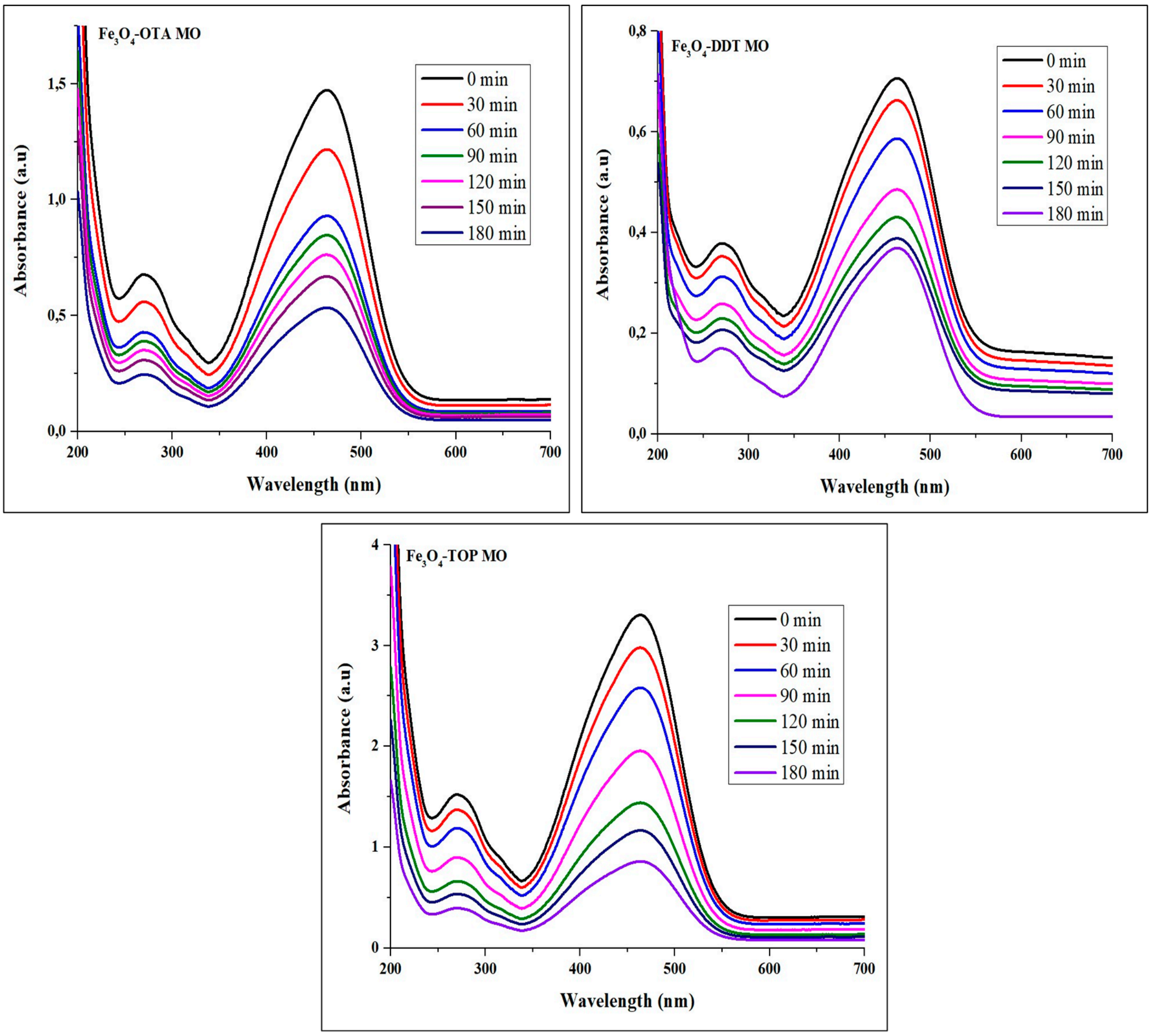

Figure S5. Absorption spectra of methyl orange (MO) over (a) Fe_3O_4-OTA, (b) Fe_3O_4-DDT and (c) Fe_3O_4-TOP nanoparticles

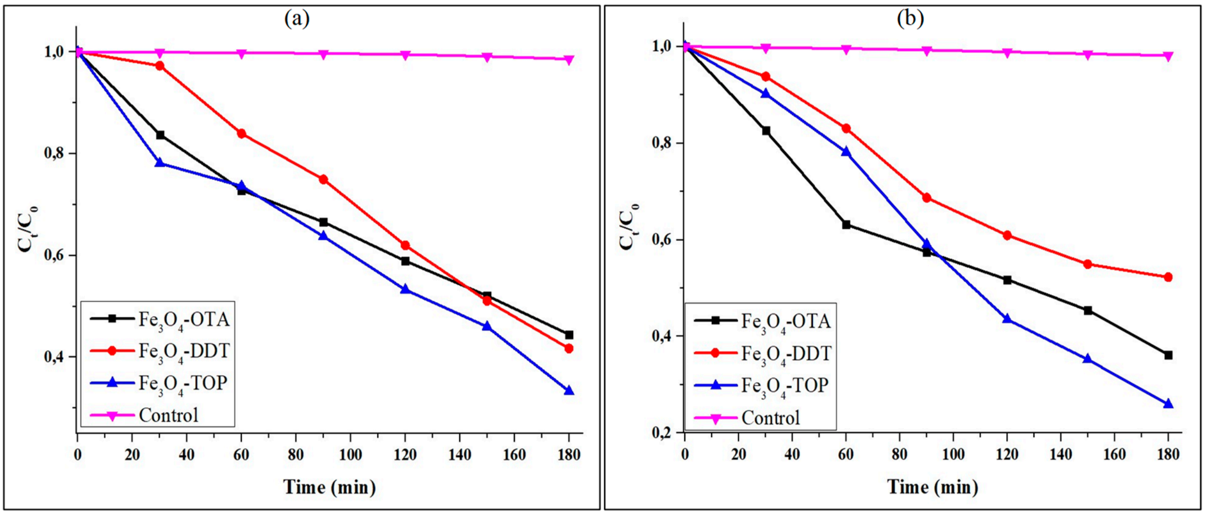

Figure S6. (a) MB and (b) MO C_t/C_0) versus irradiation time plots.

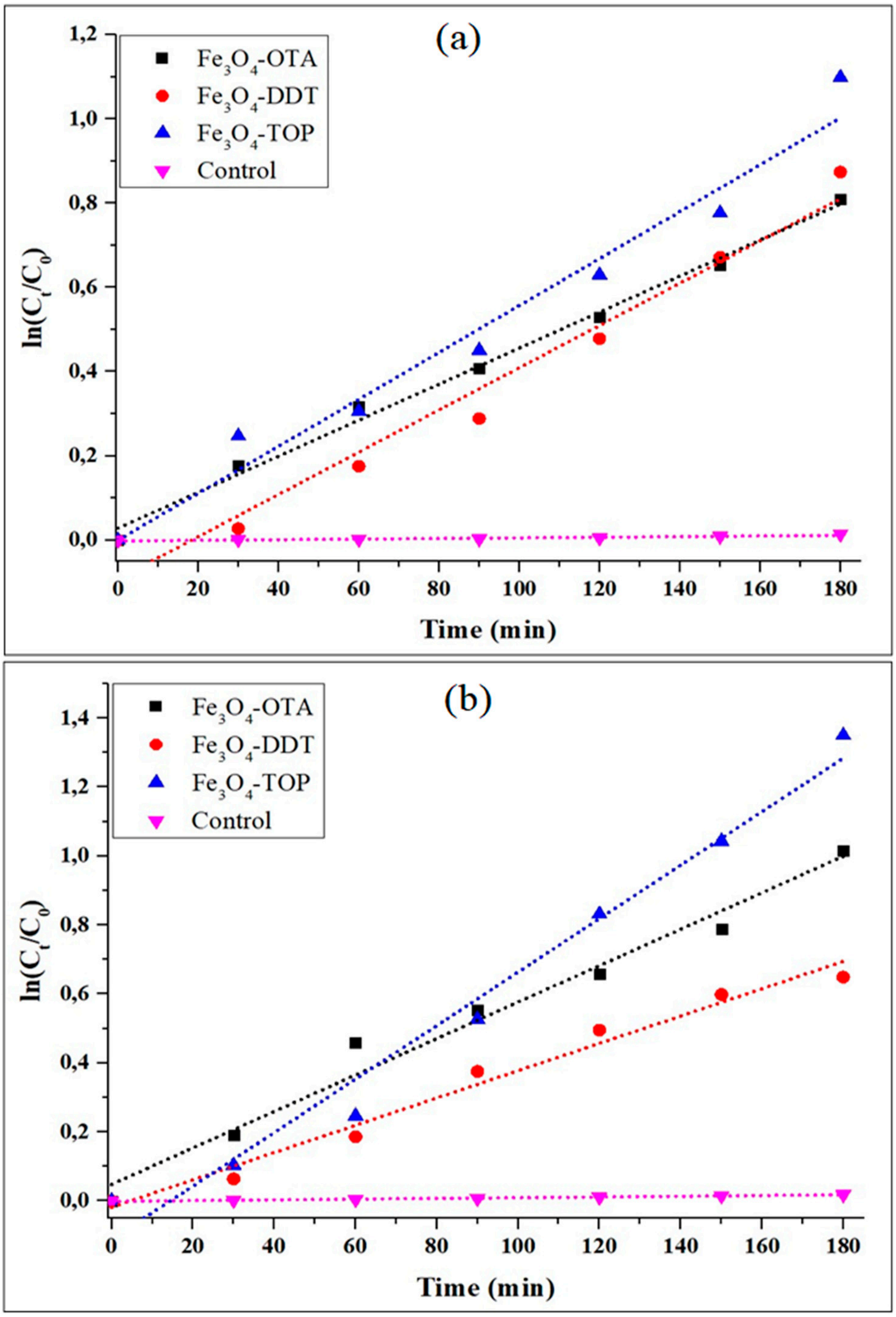

Figure S7. Methylene blue (a) and methyl orange (b) photocatalytic degradation kinetics using iron oxide nanoparticles as photocatalysts.

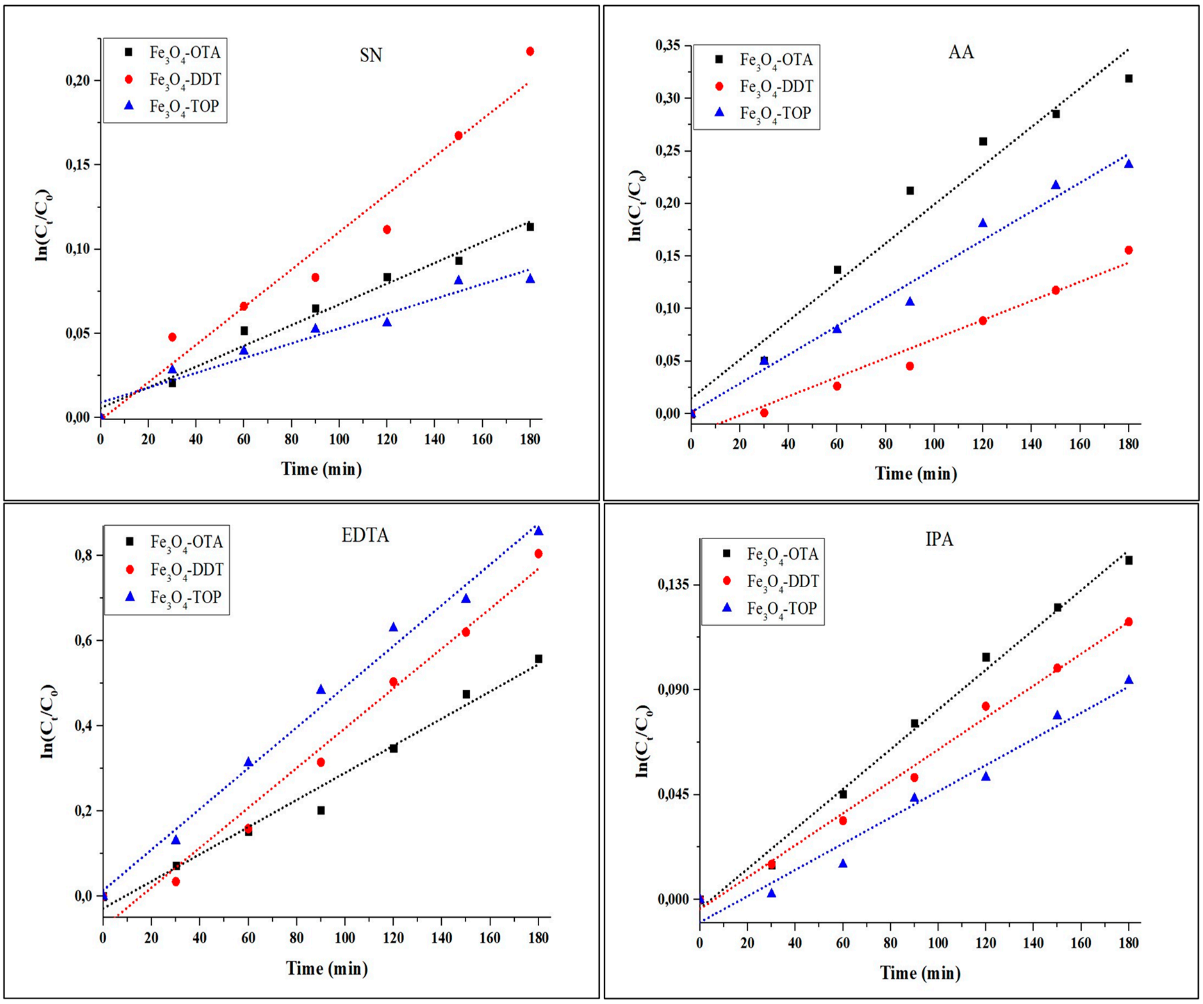

Figure S8. Photodegradation kinetics plots of MB by iron oxide nanoparticles in the presence of scavengers

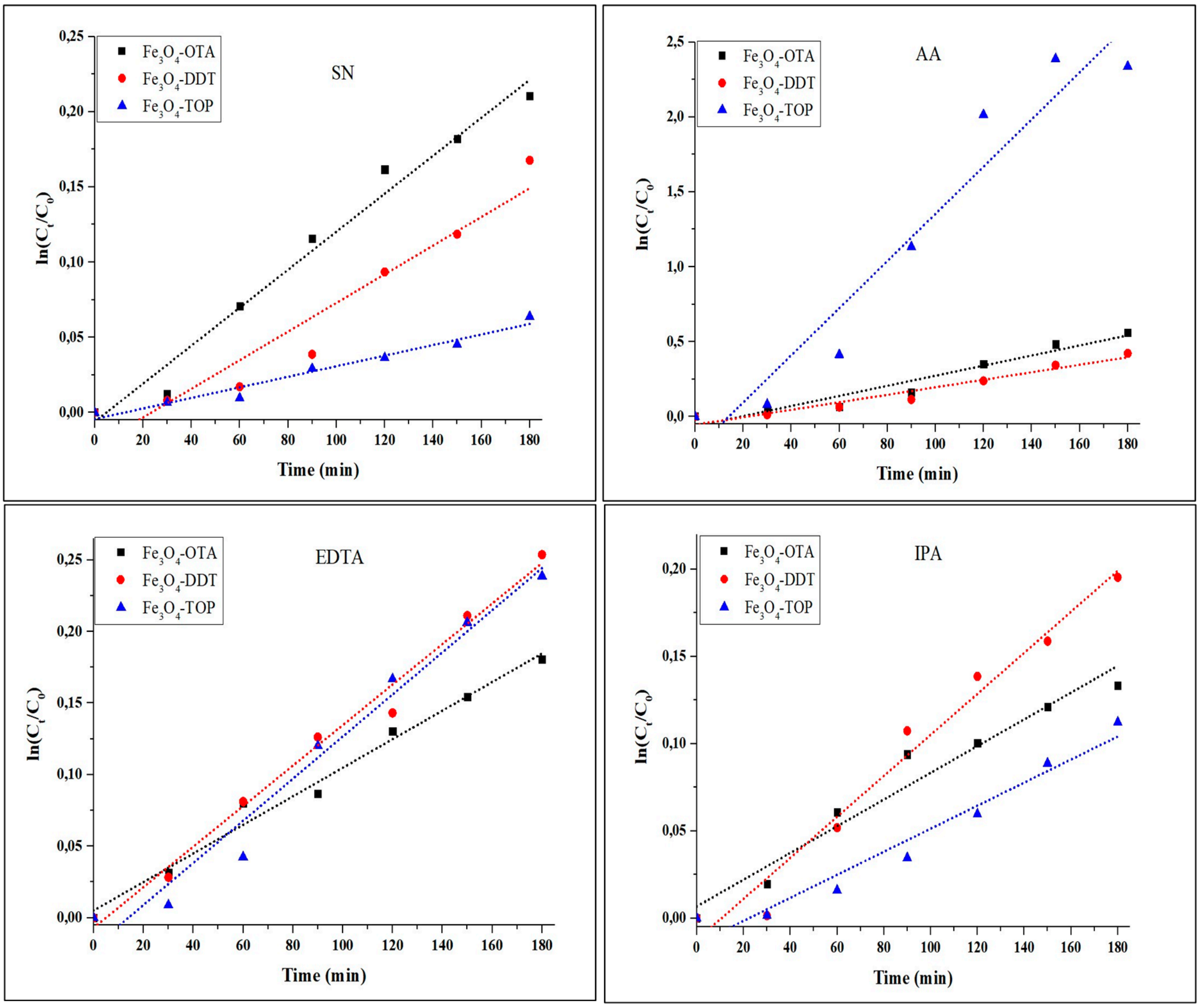

Figure S9. Photodegradation kinetics plots of MO by iron oxide nanoparticles in the presence of scavengers
